# Traditional medicinal knowledge and practices among the tribal communities of Thakht-e-Sulaiman Hills, Pakistan

**DOI:** 10.1186/s12906-021-03403-1

**Published:** 2021-09-13

**Authors:** Khalid Ahmad, Mushtaq Ahmad, Franz K. Huber, Caroline S. Weckerle

**Affiliations:** 1 Department of Environmental Sciences, COMSATS University Islamabad, Abbottabad Campus, Abbottabad, Pakistan; 2 Department of Plant Sciences, Quaid-i-Azam University Islamabad, Islamabad, Pakistan; 3 Institute of Systematic and Evolutionary Botany, University of Zurich, Zurich, Switzerland

**Keywords:** Ethnobotany, Traditional knowledge, Tribal communities, Thakht-e-Sulaiman Hills

## Abstract

**Background:**

Little is known about the medical material and practices of tribes in the western border areas of Pakistan. The local population has inhabited this remote and isolated area for centuries, and gained medicinal knowledge with personal experiences and knowledge learned from forefathers. Due to the geographical isolation of the communities in the Sulaiman hills of Pakistan and their unique culture, the area is of importance for exploration and assessment.

**Methods:**

A total of 116 informants were interviewed in five foothill villages and the associated migratory mountain villages during 2010–2012 and 2015. Information was gathered mainly through semi-structured interviews and freelisting. Local diseases were categorized based on symptoms and affected organs. Descriptive statistics were used for data analysis.

**Results:**

Depending on the type of illness, typically a pulse diagnoser or a religious specialist is consulted. Medicinal plant knowledge and use is mostly known and advised by elders within the family. A total of 44 plant species from 32 families (588 use reports), 7 animal species and 6 minerals and other sources (384 use reports) were documented as materia medica. Among the plants, the Lamiaceae is the most dominantly used plant family, followed by Pinaceae. The most frequently reported single species was *Teucrium stocksianum*. The most often mentioned diseases and treatments fall into the categories of gastrointestinal, ritual, and musculoskeletal diseases. The use of goat and sheep skin as medicine was pivotal in the local medicinal system. Remedies from animal parts and other biological and non-biological sources were mainly used for musculoskeletal ailments and ritual treatments. Overall, people rely on both traditional and biomedical medication and treatments and combination of these systems.

**Conclusion:**

This paper provides insight into the pluralistic medication system of rural communities of northwest Pakistan. It highlights the materia medica most commonly in use. A considerable part of the documented materia medica and local practices is part of an oral tradition and cannot be found in written sources or scientific articles. The gaining of new medicinal knowledge in the area was the good sign of continuation of traditional medicinal practices.

## Background

In rural areas, the local materia medica typically consists of 5–30% of the plant species of the available flora [1–3]. Further, animals and minerals are also used as medicine [4]. Local medicinal knowledge and traditional practices are heavily impacted by acculturation and modernization processes, and, if left undocumented, valuable knowledge may get lost for future generations [5]. Traditional health care systems, complemented with western medicine, are widely considered as viable solutions for improving human health in rural areas of developing countries worldwide [6].

Traditional medicinal practices in Pakistan have a long history and are largely based on the Unani Tibb, the Greco-Arab system of medicine. The Unani system relies on the concept of humours and aims for nature and mankind to coexist in a balanced manner. Unani Tibb traces its origins back to Hellenistic Greece. It was later adopted by the Arabs, and was extended to both Europe and Asia. Chinese and Indian medicine enriched it further. It proliferated in India under the Muslim rulers around 1350 AD [7, 8]. Unani is still significant in Pakistan especially among tribal peoples where it is considered as first line treatment [9–11].

Traditional medicine has been accepted and integrated into the national health system of Pakistan [12]. Professional practitioners must be registered by their respective councils, i.e., the National Council for Unani Tibb and the National Council for Homoeopathy. There are approximately 50,000 registered Hakims/Tabibs (Unani medicine practitioners), 6000 Homoeopaths, 537 Vaids (Ayurveda medicine practitioners), about 28 recognized Tibbia colleges and two Universities in the country [13, 14]. About 457 Tibbi dispensaries and many private clinics provide medication publicly throughout the country with 300–350 Unani and around 300 homoeopathic manufacturing companies producing drugs [10, 12, 15].

Traditional medicine in Pakistan is popular due to its affordability, availability and accessibility [10]. Around 63% out-of-pocket expenditure of the public is for health issues, and costs tend to be a major barrier in pursuing suitable health care [16]. The government spends about 2.6% of GDP on health [17], and their primary health care services play a negligible role in country-side areas. Thus, people in remote areas of Pakistan rely heavily on traditional medicines.

A literature review shows that the majority of ethnomedicinal studies in Pakistan are centered on the Himalaya range [18, 19], some studies are reported from the Karakoram [20–22], Hindu Kush [23, 24] and Salt ranges [25, 26], while remote tribal areas of the Sulaiman Mountains are neglected. The present study, therefore, aims to investigate and document the traditional medicinal knowledge and approach, and the materia medica among a tribal community in the Thakht-e-Sulaiman hills. This may contribute to our understanding of the medicinal pluralism and traditional use of materia medica in the countryside of Pakistan.

## Methods

### Study area

**Fig. 1:**
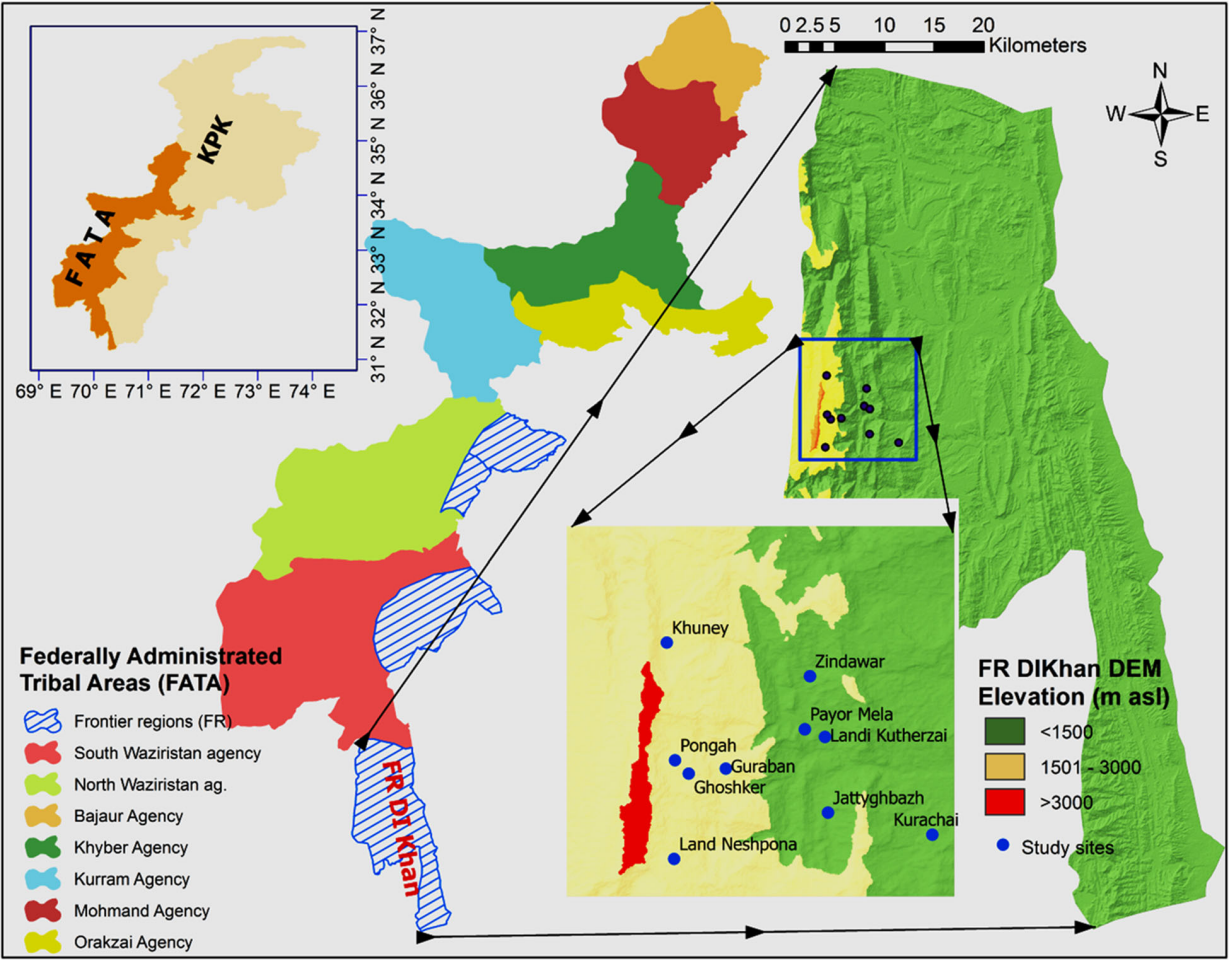
Location map and the Digital Elevation Model (DEM) of the study area showing Province Khyber Pakhtunkhwa, ex-FATA with F.R.D.I. Khan and the elevation classes. DEM data was downloaded from the online data portal (URL https://earthexplorer.usgs.gov/) of the United States Geological Survey. Elevation classes and map were developed using ArcGIS 10.5 software

Field research of about 24 months was conducted by the first author from 2010 to 2012 and in 2015 in the tribal areas of northwest Pakistan, the then called Federally Administered Tribal Areas (FATA)- recently (in 2018) merged with province Khyber Pakhtunkhwa. We focused on tribal communities in the very south of FATA, living on the eastern side of the Thakht-e-Sulaiman (**تخت سلیمان**) Mountain, the highest peak (3450 m) of the area (Fig. 1). The foothill area ranges from arid to semi-arid, with 200-500 mm precipitation mostly in July–August and December–January [27]. Summer starts in May and lasts until September with a mean daily maximum of 40 °C, and winter lasts from November to March with a daily maximum of 5.7–7.6 °C. The weather is generally warmer on the eastern side of the mountain. Vegetation changes from dry sub-tropical to dry temperate from east to west with increasing altitude. The top of the Thakht-e-Sulaiman is covered with coniferous forests [28].

The research area belongs to the Frontier Region of Dera Ismail Khan (F.R. D.I. Khan), with a total area of 2008 km^2^ and a population of approximately 68,556 [29]. Two tribes live in this area, the Sherani (شیرانئ) in the west and the Ustranas (اؤسترانئ) in the east [30]. The Sherani area is divided into plains and hills. This study focuses on the hills of the Sherani area which are inhabited by three sub-tribes: the Oba Khel- اوباخیل (ca. 18% area), Hussan Khel- حسن خیل (20%) and Chual Khel- چؤل خیل (12%). Interviews were conducted among the Sulthan Zai (سلطان زئ) sub-tribe of Oba Khel, which lives in an area of approximately 145 km^2^ [31].

Interviews were conducted in five foothill villages i.e., Payor Mela, Landi Kutherzai, Zindawar, Jaty Ghbazh and Kurachai (1). پئوڑمیلا, (2). لنڈئ کؤتڑزئ, (3). زندہ وا ر, (). جٹئ غبژ, (1). کوڑاچائ) between 1000 and 1200 masl and the associated ‘migratory villages’ in the mountains between 2300 and 2600 masl, where some people migrate in summer with their herds (Fig. 1). Livelihood strategies include livestock raising, timber cutting, non-timber forest products collection and labor work on daily wages. The informants’ details, demographic situation and brief description of foothill villages are shown in Table 1 (for migration and other details of the study sites; see [28, 31].

**Table 1:** Comparison of demographic situation and description about each foothill village

Village name	Payor Mela	Landi Kutherzai	Zindawar	Jatty Ghbaz	Kurachai
Village size (No. of families)	36	55	21	42	25
Sample size (No. of informants)	22	35	11	25	12
Informants age (mean ± SD)	43.54 ± 16.38	39.72 ± 15.94	45 ± 15.11	39.65 ± 12.38	35.83 ± 9.73
Number of family members (mean ± SD)	14.92 ± 5.64	12.44 ± 6.65	15.67 ± 6.38	14.75 ± 6.25	10 ± 2.48
Migration ratio (%)	50 (to mountains)	61 (to mountains)	50 (Within foothills)	38 (Within foothills)	0
Bilingualism (%)	54	25	1	24	8
Average/month/head expenditure (in PKR; ±SD)^1)^	931 ± 212	1063 ± 370	941 ± 194	1166 ± 537	1420 ± 457
Dependency on livestock as a source of income^2)^	75%	64%	87%	55%	67%
Dependency on Non-Timber Forest Products (NTFPs) as a source of income^2)^	24%	39%	25%	24%	8%
Elevation (meters above sea level)	1116	1052	1146	1155	1184
Population density	Moderate	Densely populated	Less populated	Moderate	Densely populated
Vegetation cover	Moderate	Thin vegetation	Dense vegetation	Above moderate	Thin vegetation
Soil type	Mostly stony	Clay soil	Diverse types of soil	Mostly stony	Totally stony

1) 1 PKR = 0.009527 USD (December 31, 2015)

2) 75% dependency on livestock means that 75% of the households depend on livestock as their main income source. Similarly, 24% dependency on NTFPs mean that 24% of households depend on selling NTFPs as main income source

### Interviews

A total of 116 informants between 20 and 100 years with an average age of 41 (SD ±13) years was interviewed. In the first phase, unstructured and semi-structured, formal and informal in-depth interviews (*n* = 58) [32] were conducted with informants in the local language, Pashtu. Also, personal observations and group discussions were held to get an overview of general concepts of natural phenomena and familiarize with local terms and their emic definitions [33]. In the second phase, detailed interviews were conducted with local health care specialists for understanding the local health care system in the area (*n* = 11). In the third phase, successive oral freelists were performed with individuals of each village to check for completeness of the collected knowledge as well as its variation at individual and community level (*n* = 47). Transect walks were made along the villages with key informants both at foothills and mountainous areas for specimens’ collections and triangulation of data. Most informants were male due to cultural restrictions on involving female informants. Key female informants were included indirectly, mostly through interviewing their sons.

We built rapport with the local communities and were allowed to live with the people, accompany them during their daily life, and attend ritual ceremonies. The ethical guidelines of the International Society of Ethnobiology [34] were strictly followed during the whole research process. Consent was obtained from every informant before interviewing where objectives, procedure and methodology of the project were also explained.

### Plant specimens

Specimens were prepared of all documented plants (Table 5). These were identified by taxonomists at Quaid-I-Azam University Islamabad Pakistan (by Dr. Zahid Ullah & Dr. Mushtaq Ahmad) and reconfirmed by comparing with specimens in the Herbarium of Pakistan and the Flora of Pakistan [45, 46]. Families were assigned according to Chase et al. [47] and species names were cross checked with *The Plant List* [48]. All voucher specimens with accession numbers were deposited at the Herbarium of Pakistan (ISL), Quaid-I-Azam University Islamabad Pakistan.

### Data analysis

Information on medicinal plants was analyzed using use reports (URs- as in Table 3, 4, 5). One use report corresponds to a specific plant part administered in a specific way against a disease as mentioned by one informant. Freelist data were analyzed using descriptive statistics. To determine the most frequently used plant species for treating a particular ailment category by the informants of the study area, we calculated the fidelity level (FL-, as in Table 6) by following Alexiades [49]. The availability of species was categorized into frequent, occasional, and rare based on personal observations in the field and discussions with the informants by following the criteria of DAFOR scale. Local terms for diseases were reconfirmed with the regional medical doctors. To facilitate cross-cultural comparisons and to highlight uniqueness and similarities, we categorized all the diseases mentioned by the interviewees into 16 disease categories according to the symptoms they cause and the organs they affect [50]; see Table 2).

**Table 2:** Disease categories based on symptoms and the affected organs

Symptoms	Disease category
Reduces the duration of Typhoid, body coolness, Sun stroke, Sun shock, influenza, General body ailments, piles, diabetes	Multisystem
Applied on small external wounds, to dry them, Papillae, blisters, abscess, pustules, skin allergy, powdered is spread in joints of kids, applied on skin after plant splinters and spines get penetratied and cause injury	Dermatological
Stomach ailments/problems, abdominal/stomach gases and pain, constipation/dysentery, diarrhea/loose motion	Gastrointestinal
Internal injuries and wounds, bones fractures, sprains, strains	Musculoskeletal
‘Voice infection’, unusual diseases, *masiyath,* i.e., diseases caused by spirits (Jinn), evil eye	Ritual
Teeth diseases, insects in the teeth and teeth bleeding	Dentistry
High temperature, malaria	Fever
Eye irritations/troubles	Ophthalmological
Spermatorrhoea	Urenogenital diseases
Cough	Respiratory
Jaundice, Hepatitis	Hepatic
Used against flatulence, obesity	Obesity
Low and high blood pressure	Blood system
Snakes didn’t enter your home, repel mosquitos	Repellent
Used for better health, nutritious, tonic	Medicinal food
Pain in ear	Earache

## Results

### Local healthcare system

The local health care system is pluralistic and consists of different types of specialists with different backgrounds. According to their needs, local people visit one or several of these specialists. The choice of medicine is based on severity of disease, effectiveness of the medicine and ease of availability. Wearing the skin of a goat/sheep is usually applied in severe cases, medicinal plants are used for moderate ailments, and minerals and other animal products are used for specific ailments or as alternatives if plants are unavailable.

Two types of ritual specialists are found: a) Mullayan (ملایان), the religious specialists with formal religious education from a religious school called Madrasa (مدرسہ). He uses religious knowledge for healing including the scripture from the holy books and practices from Quran, Hadith, and Sunnah; and is responsible for other religious duties like collective prayers and funeral processions. People visit Mulla usually in cases of ‘masiyath’ (ماسیت)—a group of ailments including any unusual disease believed to be caused by spirits (Jinn), or other soul related illnesses with symptoms like neurological or psychological trauma/disorders, hypervigilance and pervasive feelings of terror—which are treated with ‘ta’wiz’, dam and du’a’- (تعویذ، دم، دعا), i.e., amulets, blessings, and prayers. The amulets typically consist of a piece of paper with holy inscription wrapped in a cloth and/or leather and hung/fastened around the neck or arm. Most villages have a Mulla and they tend to have average medicinal plant knowledge; all Mulla we met were men, but nowadays women can also attend religious schools. b) Aamel (عامل), is a ritual specialist consulted when people contract unusual diseases related to spirits. They work with rituals which may last up to 40 days. Usually, they are male but female Aamel also exist. Aamel apprentice with experienced Aamel and go through arduous training. They communicate with spirits, locally called Paerai (پیرائی), and are widely known as Jinn. These are entities which can cause physical and mental harm to humans but can be tamed by Aamel. These specialists are rare in the area, and usually lack knowledge of medicinal plants.

Consultants or pulse diagnosers identify the ailment through pulse and provide or suggest appropriate treatment with medicinal plants or other drugs, wearing of animal skin, ritual treatment, or biomedical treatment. The consultant’s knowledge about pulse is considered as a gift of God which is mostly transferred from generation to generation (usually male). Usually, each village has a consultant/pulse diagnoser. Locals visit consultants when unaware of what ails them. Consultants have good medicinal plant knowledge.

Traditional bone setters adjust broken and disjointed bones. They are rare in the area and their knowledge is inherited from elders. Rural bone setters tend to use medicinal plants whereas urban bone setters use conventional pharmaceuticals.

Traditional midwives or birth attendants - usually one per village - are wise and experienced women invited during childbirth. They are knowledgeable regarding medicinal plants. Biomedical drugs are administered by medically trained doctors in clinics in urbanized areas. Untrained drug sellers are found in every 3rd or 4th village. They also advise usage and administering of drugs.

There are no specific local medicinal plant specialists, but elders have knowledge of medicinal plants and use them in their families. They are mostly old and experienced people who inherit this knowledge from ancestors and other elders.

### Local therapeutic concepts and treatments

The local materia medica is composed of plants, animals, minerals, and other sources while the local etiology revolves around the Unani concept of humours. This concept reached the area through practitioners trained in Unani medicine from united India who settled in remote tribal areas for community services. Their knowledge was incorporated into the knowledge system of the communities (personal communication with Unani medicine experts).

The concept of “bitter” is locally associated with medicine and an often-used proverb says: “everything bitter except poison is good for health while everything sweet except honey is harmful”. ‘Voice infection’ (ژاغ) is another local concept related to health and disease. It assumes that some people’s voice has naturally an infectious effect on the patient or his/her wounded body parts. It can happen intentionally or unintentionally. Beside plants, gold or silver is preferentially used to cure or prevent the effect of ‘voice infection’ (Table 3).

**Table 3:** Other animal and mineral remedies

Species/ Entity	Common name/Local name (in Pashtu language)	Habit	Status	Disease category	Parts Used	Use	URs (Informants)
Gold	Gold/ *Sra zer*	Mineral	Precious metal, wear by women taken from market	Ritual	Gold	Gold is used directly or put in water for some time and that water is mixed with flour to make dough which is wrapped in cloth and fastened around (Plate 1; E) or near the infected organ against ‘voice infection’. It is commonly used after the circumcision of male child against ‘voice infection’.	41 (41)
*Gallus gallus domesticus*	Chicken egg/ *agayi*	Bird	Domestic	Musculoskeletal	Egg	It is taken directly or in half boiled condition for bone healings. Sometimes *curcuma longa* rhizome powder is mixed with it.	33 (33)
mineral-pitch or mineral-wax	mineral-wax*/ Salajeeth*	Mineral	Secreted from rock cracks and locally collected	Musculoskeletal	Mineral-wax	It is sticky tar-like substance of brown color which is secreted from rocks called mineral-pitch or mineral-wax. It is eaten directly but sometimes taken with milk against musculoskeletal ailments.	28 (28)
Brass coin	Brass coin/ S*orvay paysa*)	Alloy	Previous currency rarely available in homes	Ritual	Brass coin	It is one cent of 1950s–60s Pakistani currency. A hole is made in the mid of it and fiber is passed though it which is then fasten around infected organ against ‘voice infection’.	23 (23)
Marketed mineral stone	Mineral stone/ *Da nazer dana*	Mineral	Small size stones of red-orange color available in markets	Ritual	Mineral stone	It is wrapped in cloth and fastened around or near the infected organ as Amulet, against ‘voice infection’. It is rarely available in the market.	18 (18)
A specific common stone	common stone/ S*heen kanrai*	Mineral	Locally available near/around water channels	Dermatological	Stone powder	Crashed into pieces and sieved through cloth to obtain powder which is spread on small wounds on the body for rapid healing.	17 (17)
*Capra falconeri*	Sulaiman Markhor/ *Gharchanay*	Animal	Wild	Hepatic	Bile of gall bladder	The fresh bile of gall bladder is taken orally against hepatitis B and C. Its meat is also considered good for health.	14 (12)
*Gallus gallus domesticus*	Chicken soup/ *Yakhni*	Bird	Domestic	Musculoskeletal	Meat	It is boiled in water with black pepper powder and the decoction (chicken soup) is taken orally for bone healings.	6 (6)
*Erethizon dorsaum*	Porcupine*/* *Skonr*	Animal	Wild	Musculoskeletal	Body fats	The fats oil is applied on affected organ.	4 (4)
*Equus africanus asinus*	Ass/ *khra*	Animal	Domestic	Respiratry	Milk	Fresh milk of ass is taken directly against whopping cough.	3 (3)
Silver	Silver/ *Speen zer*	Mineral	Precious metal, wear by women taken from market	Ritual	Silver	The silver is wrapped in cloth and fastened around or near the infected organ against ‘voice infection’. Mostly when gold is not available then silver is used.	3 (3)
*Ursus americanus*	Black bear/ *Yaaz*	Animal	Wild	Musculoskeletal	Body fats	The fats oil is applied on affected organ.	3 (3)

Evil eye is a concept that describes the power of envy and jealousy. Humans obsessed by envy or jealousy can, with their eyesight, harm their fellow men intentionally or unintentionally. A person with evil eye can harm even when they are pleased to see someone or somebody’s possessions. Children, mothers during pregnancy before and after childbirth, ill/injured as well as healthy and wealthy people are vulnerable to it. For protection, a religious amulet is mostly used, or sometimes a small temporary black scar is made with coal on the visible part of the skin.

Wearing of goat/sheep skin is locally considered a paramount medicinal tool. It is used for a large number of ailments especially in cases of emergency and complications (Table 4). Generally, goat skin is considered cold and advised to be worn in summer, while sheep skin is warm and is advised for the winter. Use of each can also be advised regardless of the season. According to key informants, use of the skin needs special care, otherwise, the disease may worsen. Wearing the skin needs to be carefully adjusted to the progression of the disease. For example, skin wearing is advised only at the beginning of malaria; advised at the beginning or end of typhoid but not at the climax when symptoms are the strongest. Correct and effective usage is mostly advised by the consultants of the area or by the elders of the family. It is believed that an imbalance of hot/cold usually causes common illnesses. Wearing of the skin influences this balance, smoothens and supports the body according to its requirements, and detoxifies through suction.

**Table 4:** The use of goat and sheep skin

Animal	Disease category	Description	URs (Informants)
Sheep	Musculoskeletal	It is used for all types of Musculoskeletal problems due to falling from high place, get strong hits during fights etc.	45 (45)
Sheep	Musculoskeletal	Women wear it after childbirth as it finishes the pain and heal the internal injuries and wounds.	40 (40)
Sheep	Hepatic	When the yellowness of the skin gets high then the skin of sheep is used, or it is used to remove the remaining symptoms of hepatitis when the patient gets recently recovered.	21 (21)
Goat	Fever	At the start of malaria, but not after getting 2–3 days old as then it may convert it to typhoid and get complicated.	18 (18)
Sheep	Multisystem	Typhoid but not at the start of it, either towards its end or after it get finished then to finish its affects in the body.	11 (11)
Goat (in summer), Sheep (in winter)	Musculoskeletal	Against general pain in the body or bones which is not getting better since long time.	10 (10)
Goat	Fever	Used against fever at its beginning stage.	9 (9)
Goat (in summer), Sheep (in winter)	Fever	Against fever when it gets complicated or when it does not finish with normal medication, or when body temperature gets very high or reach to head, or to finish hidden fever.	8 (8)
Goat	Multisystem	In summer, it is used against sunstroke.	7 (7)
Goat	Multisystem	Used for body cooling when feel extensive hotness inside the body.	6 (6)
Goat (in summer), Sheep (in winter)	Multisystem	General body ailments, i.e., to get the weak person’s body stronger, increase resistance against diseases, finish the symptoms of disease.	4 (4)
Goat (in summer), Sheep (in winter)	Ritual	Black colored skin is to be worn in case of some unusual diseases, e.g. caused by spirits (Jinn).	3 (3)
Goat (in summer), Sheep (in winter)	Dermatological	When bitten by a large number of honeybees (usually honeybees’ bites in summer).	3 (3)
Goat	Gastrointestinal	Against acute diarrhea.	2 (2)
Goat (in summer), Sheep (in winter)	Dermatological	Against acute skin allergy.	2 (2)
Goat (in summer), Sheep (in winter)	Urinogenital	When menstruation gets abnormal.	2 (2)

Other remedies made from animals include bear and porcupine fats used against musculoskeletal problems, ass milk against whopping cough, and gall bladder bile from the Sulaiman Markhor—a wild goat (*Capra falconeri*)*—*against hepatitis B and C (Table 3). Minerals like gold, mineral stone, silver or brass coins are often ritually used for protection against ‘voice infection’.

### Diversity and use of medicinal plants

**Fig. 2:**
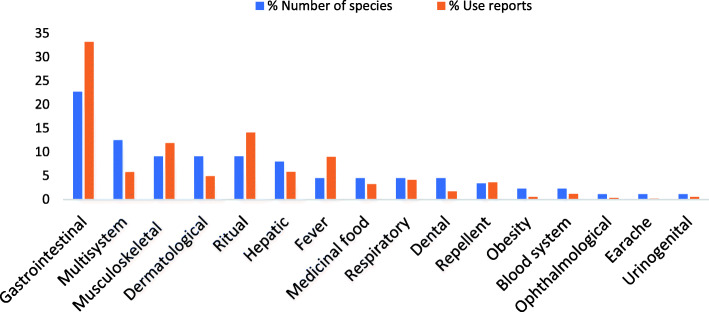
Medicinal plants used in various disease categories

**Fig. 3:**
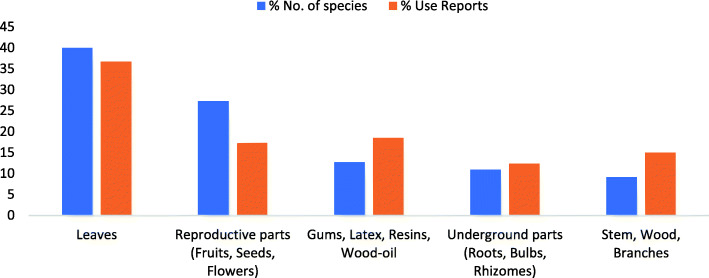
Plant parts used as medicine

A total of 44 species of plants were documented with 588 use reports (Table 5). The medicinal species are herbs (21 spp.), trees (12 spp.), shrubs (9 spp.), and climbers (2 spp.) which are distributed among foothills (28 spp. with 258 UR) and mountainous areas (16 spp. with 330 UR; Table 5). Among all, a single species (*Curcuma longa*) is cultivated, three are semi-cultivated, i.e., present both in wild and cultivated form (*Ficus palmata*, *Olea ferruginea*, *Punica granatum*), while the remaining are wild. Majority of the species are frequently available (23 spp.) while some are occasional (16 spp.) and others rare (5 spp.). Almost half of the plants (28 spp.) are used only in fresh form while the remaining (16 spp.) are used in both fresh and dried forms. Additionally, 16 species among the documented medicinal plants are also used as food (Table 5). All the diseases cured with medicinal plants are categorized into 16 disease categories in which gastrointestinal diseases are the most commonly mentioned (with high number of species and use reports) followed by multisystem and ritual, respectively (Fig. 2). Leaves are mostly used, while gums, resins, latex, and wood-oil also play an important role (Fig. 3).

**Table 5:** Medicinal plant characteristics and use for different ailments

Species with Authority citation, Accession no. and Family	Local name / Habit	Occurrence / Availability	Disease category with UR	Route of application / Used form	Parts Used / Mode of preparation	Use	Reported from Pakistan	Used as food	URs (Informants)
*Acacia modesta* Wall. (127146) **Fabaceae**	*palous***/** Tree	Foothills **/** Frequent	Multisystem (1), Medicinal food (2), Musculoskeletal (11)	Oral **/** Fresh & dried	Terminal buds (1), Gums (13) **/** Without processing	Young fresh twigs are chewed for body coolness. Gums are eaten directly for musculoskeletal problems. Also, the gums are given to women as tonic after childbirth. **Food:** The gums are eaten as food.	[19, 35]	No	14 (12)
*Alternanthera paronychioides* A.St.-Hil. (127218) **Amaranthaceae**	*Penderwash***/** Herb	Foothills **/** Frequent	Ritual (2)	Topical **/** Fresh	Arial parts **/** Without processing	Arial parts are fastened around the infected organ against ‘voice infection’	“No”	No	2 (2)
*Achyranthes bidentata* Blume (127164) **Amaranthaceae**	*Jeza lashtha***/** Herb	Foothills **/** Frequent	Multisystem (1), Fever (2)	Oral **/** Fresh	Leaves **/** Decoction	Fresh leaves decoction is drunk against high temperature, malaria and sunstroke. Its use in the area is disappearing.	No	No	3 (2)
*Allium spp* L. (127217) **Alliacaeae**	*cook***/** Herb	Mountains **/** Frequent	Fever (4), Hepatic 2), Multisystem (1), Gastrointestinal (6), Musculoskeletal (1), Medicinal food (8), Respiratory (8)	Oral **/** Fresh & dried	Leaves (10), Bulb (20) **/** Without processing	The plant is eaten against cough, malaria, diabetes, hepatitis, stomach problems, warms in intestines, sprains in body, gasses in body, and also considered as better for general health conditions. **Food:** The plants is taken fresh with bread and also used as condiment.	No	Yes	30 (15)
*Prunus brahuica* (Boiss.) Aitch. & Hemsl. (127160) **Rosaceae**	*Ghorghosthai***/** Tree	Foothills **/** Occasional	Repellent (14), Musculoskeletal (2), Dermatological (4)	Oral & topical **/** Fresh	Gums (6), Tertiary branches (14) **/** Without processing	It is strongly believed in the area that if have the stick of this in your hand or home then snakes do not bite you and do not come in your house. Gums are mixed with sugar and then eaten for musculoskeletal problems. Also gums are applied on abscesses.	“No”	No	20 (16)
*Arisaema jacquemontii* Blume (127174) **Araceae**	*Kharpata***/** Herb	Mountains **/** Occasional	Ritual	Oral **/** Fresh	Fruit (1), Root (1) **/** Without processing	Its root and fruit are eaten fresh in a small amount which finish the “*masiyath*” disease.	“No”	No	2 (2)
*Berberis calliobotrys* Bien. ex Koehne (127222) **Berberidaceae**	*thor korai***/** Shrub	Mountains **/** Occasional	Musculoskeletal (24), Hepatic (1), Urinogenital (1), Blood System (1)	Oral **/** Fresh	Roots / Paste	Fresh root bark is taken paste is orally against musculoskeletal ailments, high blood pressure and spermatorrhoea (but excessive use and touch with teeth can cause loss of teeth). The decoction of root bark is taken against jaundice.	No	No	27 (24)
*Bergenia ciliata* (Haw.) Sternb. (127297) Saxifragaceae	*da ovr sawai***/** Herb	Mountains **/** Rare	Dermatological	Topical **/** Fresh	Leaves **/** Ash	The ash of the leaves is applied on fire burnt wounds.	No	No	1 (1)
*Calotropis procera* (Aiton) Dryand. (127228) **Apocynaceae**	*spalmai***/** Shrub	Foothills **/** Frequent	Dermatological (9), Ritual (16)	Topical **/** Fresh & dried	Latex (22), Stem (3) **/** Juice (1), Without processing	Milky latex is applied on external wound and on Abscess. Latex is applied on external wound or its wood part (fresh or dried) is fastened around the wounded/infected organ against “‘voice infection’”.	[26, 36]	No	25 (23)
*Caralluma tuberculata* N.E. Br. (127133) **Apocynaceae**	*Pamanai***/** Herb	Foothills **/** Rare	Blood system (2), Respiratory (4), Multisystem (14), Earache (2), Gastrointestinal (9), Medicinal food (6)	Oral & topical **/** Fresh	Arial parts **/** Juice (1) Without processing (36)	A single drop of its extract is dropped in the painful ear of kids. Eaten raw or cooked which is used against blood pressure, piles, diabetes. Arial parts are warmed on fire and eaten for cough. Eaten against intestinal warms, for stomach problems, gasses in the body and considered as better for health. **Food:** This is very famous wild vegetable in the area.	[36, 37]	Yes	37 (12)
*Celtis australis* L. (127176) **Cannabaceae**	*thaghah***/** Tree	Foothills **/** Occasional	Ritual	Topical **/** Fresh & dried	Branches **/** Without processing	Fresh or dried young branches are used as Amulet (kept in the cloths or fasten around the arm) against evil eye.	No	No	3 (3)
*Citrullus colocynthis* (L.) Schrad. (127182) **Cucurbitaceae**	*Kandelay***/** Herb	Foothills **/** Frequent	Dental	Incense **/** Fresh & dried	Seeds **/** Smoke	Fresh and dried seeds are put on the hot coal and the smoke is directed to the infected teeth (i.e. against worms in the teeth).	No	No	3 (3)
*Clematis orientalis* L. (127139) **Ranunculaceae**	*chengenwaly***/** Climber	Mountains **/** Occasional	Dental	Topical **/** Fresh	Leaves **/** Paste	Fresh leaves paste is kept on arm for short time (as longer keeping make wound on arm) against teeth pain.	No	No	5 (5)
*Cotoneaster microphyllus* Wall. ex Lindl. (127234) **Rosaceae**	*manray***/** Shrub	Foothills **/** Frequent	Gastrointestinal	Oral **/** Fresh	Fruit **/** Without processing	Fresh fruits are taken orally against constipation. Not often used as medicine. **Food:** Also used as common wild fruit.	No	Yes	3 (3)
*Curcuma longa* L. (127235) **Zingiberaceae**	*kurkaman***/** Herb	Foothills **/** Frequent	Musculoskeletal (8), Hepatic (8)	Oral & topical **/** Fresh & dried	Rhizome **/** Without processing (8), Powder (8)	Dried rhizome powder is mixed with half boiled egg or taken with water for Musculoskeletal ailments. The rhizome is hanged around the neck against Jaundice. This plant is cultivated, and the locals bought it from the market. **Food:** It is commonly used as condiment.	[38]	Yes	16 (12)
*Cymbopogon jwarancusa* (Jones) Schult. (127141) **Poaceae**	*boyan sergarai / Herb*	Foothills **/** Frequent	Multisystem	Oral **/** Fresh & dried	Roots **/** Decoction	Root bark is boiled in water. Take 1 cup for 2–3 days, shorten the duration of typhoid.	No	No	6 (6)
*Dalbergia sissoo* DC. (127237) **Fabaceae**	*sawa***/** Tree	Foothills **/** Rare	Multisystem	Topical **/** Fresh	Leaves **/** Paste	Fresh leaves paste is applied on head for cooling. Rarely available as its growing zone is below the start of foothills.	No	No	2 (2)
*Ehretia obtusifolia* Hochst. ex A.DC. (127161) **Boraginaceae**	*maraghoney***/** Shrub	Foothills **/** Occasional	Gastrointestinal	Oral **/** Fresh	Fruit **/** Without processing	Fresh fruits are taken directly against constipation **Food:** Its fruit is also considered as famine food.	No	No	3 (3)
*Ephedra gerardiana* Wall. ex Stapf. (127150) **Ephedraceae**	*oman***/** Shrub	Mountains **/** Rare	Dental (1), Dermatological (3), Gastrointestinal (39), Musculoskeletal (1)	Oral & topical **/** Fresh & dried	Secondary & Tertiary branches **/** Without processing (1), Powder (43)	Young fresh stem is chewed for teeth bleeding, dried stem powder is taken with water for stomachache and musculoskeletal problems. Also, the powder is spread on wounds for rapid healing	No	No	44 (38)
*Euphorbia prolifera* Buch.-Ham. ex D. Don (127159) **Euphorbiaceae**	*thandawany***/** Herb	Mountains **/** Frequent	Ritual	Topical **/** Fresh	Leaves **/** Without processing	Fresh leaves are fastened around wounded spot to avoid ‘voice infection’.	No	No	42 (42)
*Euphorbia prostrata* Aiton (127239) **Euphorbiaceae**	*thandawany***/** Herb	Foothills **/** Frequent	Ritual	Topical **/** Fresh	Latex **/** Without processing	Fresh leaves are fastened around wounded part to avoid ‘voice infection’.	No	No	1 (1)
*Ficus palmata* Forssk. (127240) **Moraceae**	*injar***/** Tree	Foothills **/** Occasional	Gastrointestinal	Oral **/** Fresh	Fruit **/** Without processing	Fresh fruits are eaten against constipation. Leaves are cooked and eaten as meal which is considered good against constipation. **Food:** Used as common wild fruit and its leaves as vegetable.	[19, 35]	Yes	4 (4)
*Ipomoea turbinata* Lag. (127245) **Convolvulaceae**	*kulmovala***/** Climber	Foothills **/** Occasional	Gastrointestinal (2), Hepatic (1), Obesity (6)	Oral **/** Fresh	Stem **/** Decoction	Decoction of fresh stem is taken orally against stomach gasses and obesity, also used against jaundice.	No	No	9 (7)
*Litsea monopetala* (Roxb.) Pers. (127249) **Lauraceae**	*zyer largai***/** Tree	Foothills **/** Rare	Hepatic	Oral **/** Fresh	Stem wood **/** Decoction	Decoction of fresh wood is taken orally against jaundice.	No	No	18 (18)
*Mentha longifolia* (L.) L. (127209) **Lamiaceae**	*shamshobai***/** Herb	Foothills **/** Frequent	Fever (1), Gastrointestinal (26), Multisystem (1)	Oral & topical **/** Fresh & dried	Leaves **/** Without processing (2), Powder (4), Infusion (22)	Dried leave powder is taken with water against fever and stomach problems. Fresh leave infusion is taken orally against diarrhea, abdominal pain and for body cooling. Leaves are placed beneath sleeping pillow for typhoid.	[39, 40]	No	28 (25)
*Sideroxylon mascatense* (A.DC.) T.D.Penn. (127185) **Sapotaceae**	*gurgur***/** Tree	Foothills **/** Frequent	Dermatological (1), Gastrointestinal (1)	Oral & Topical **/** Fresh	Leaves (1), Fruit (1) **/** Without processing (1), Juice (1)	Fresh leaves paste is applied on wound against the inserted spins. Fresh fruit is considered useful against constipation, but its excessive use may cause diarrhea. **Food:** Common and well-known wild fruit of the area.	Not reported against wound	Yes	2 (2)
*Morina longifolia* Wall. ex DC. (127204) **Caprifoliaceae**	*jeaz azghai***/** Herb	Mountains **/** Occasional	Multisystem	Oral **/** Fresh	Leaves **/** Without processing	Extract of fresh leaves are taken orally for body cooling.	“No”	No	1 (1)
*Nannorrhops ritchiana* (Griff.) Aitch. (127253) **Arecaceae**	*mazarai***/** Shrub	Foothills **/** Occasional	Gastrointestinal	Oral & topical **/** Fresh & dried	Flower (1), Leaves (1) **/** Without processing	Fresh and dried flowers are taken directly for abdominal pain. Fresh leaves are fastened around the wounded organ against ‘voice infection’.	Its gums Not reported against eye irritations	No	2 (1)
*Notholirion thomsonianum* (Royle) Stapf (127166) **Liliaceae**	*shyajey***/** Herb	Foothills **/** Occasional	Gastrointestinal	Oral **/** Fresh	Leaves **/** Without processing	Fresh leaves are cooked and eaten against constipation. **Food:** Used as wild vegetable.	Yes [26, 41]	Yes	1 (1)
*Olea ferruginea* Wall. ex Aitch. (127151) **Oleaceae**	*shwawan***/** Tree	Foothills **/** Frequent	Ophthalmological (3), Dental (1), Gastrointestinal (28)	Oral & topical **/** Fresh & dried	Leaves (1), Gums (3), Fruit (28) **/** Without processing	Fresh leaves are chewed for dental problems. Gum is applied to irritated eyes with small smooth wood sticks. Fruits are eaten (fresh & dried) against constipation and it also kills intestinal worms. Also considered good for improving general health conditions. **Food:** Common and well-known wild fruit of the area.	No	Yes	32 (28)
*Peganum harmalla* L. (127154) **Nitrariaceae**	*spanda***/** Herb	Foothills **/** Frequent	Multisystem	Oral & Topical **/** Fresh & dried	Leaves (1), Seeds (1) **/** Smell (1), Smoke (1)	Fresh leaves are smashed between fingers and smelled for influenza. Smokes of dried seeds are made (by putting the seeds on hot coal) near the person suffering from *masiyath* disease. The smoke of seeds is used (by putting its seeds on water pipe) for asthma disease.	“No”	No	2 (1)
*Phlomoides spectabilis* (Falc. ex Benth.) Kamelin & Makhm. (127258) **Lamiaceae**	*zer panray***/** Herb	Mountains **/** Occasional	Dermatological	Topical **/** Fresh	Leaves **/** Infusion	Take a bath with infusion of fresh leaves against skin allergy. Its use is getting lost in the area.	No	No	1 (1)
*Pinus gerardiana* Wall. ex D. Don (127203) **Pinaceae**	*zanghozai***/** Tree	Mountains **/** Frequent	Dermatological (9), Ritual (10), Repellent (3), Medicinal food (8)	Oral & Topical **/** Fresh	Resins and wood-oil **/** Without processing	Resins are applied on wound for rapid healing. Also, resins with wood-oil^a^ are applied on wound against ‘voice infection’. The wood-oil is also applied on skin at night as mosquito repellent. **Food:** It is very famous wild fruit of the area and considered good for health as quite nutritious and warm for the body in winter.	“No”	Yes	30 (19)
*Pinus wallichiana* A.B.Jacks. (127213) **Pinaceae**	*nashthar***/** Tree	Mountains **/** Frequent	Musculoskeletal (22), Dermatological (1), Repellent (4), Ritual (7)	Oral & topical **/** Fresh & dried	Resin and wood-oil **/** Without processing	Resin is eaten directly or taken with water for musculoskeletal problems. Applied directly on wounds for healing and against ‘voice infection’. Wood-oil is also used against ‘voice infection’. The wood-oil is also applied on skin at night as mosquito repellent.	“No”	Yes	34 (29)
*Punica granatum* L. (127208) **Lythraceae**	*nergos***/** Shrub	Foothills **/** Occasional	Respiratory (5), Gastrointestinal (1)	Oral **/** Fresh & dried	Fruits **/** Paste (1), Without processing	Dried fruit is warmed on fire and eaten for cough especially in winter. Fruit is eaten fresh against constipation and diarrhea. This plant is available in the wild, but its cultivation has been started in the area. **Food:** It is well known wild fruit of the area.	Not reported for cough	Yes	6 (5)
*Rumex dentatus* L. (127196) **Polygonaceae**	*skheryai***/** Herb	Mountains **/** Occasional	Hepatic	Oral **/** Fresh	Roots **/** Decoction	Decoction of fresh root is taken orally against jaundice.	No	No	3 (3)
*Solanum surattense* Burm. f. (127157) **Solanaceae**	*maraghoney***/** Herb	Foothills **/** Frequent	Musculoskeletal (1), Obesity (1), Gastrointestinal (2)	Oral **/** Fresh & dried	Seeds **/** Without processing	Fresh or dried seeds are mixed with something sweet (due to its bitter taste) and taken orally against gasses in the body, abdominal worms, muscular pain and obesity.	[25]	No	4 (2)
*Teucrium stocksianum* Boiss. (127267) **Lamiaceae**	*da thaby botai***/** Herb	Foothills **/** Frequent	Multisystem (1), Fever (46)	Oral **/** Fresh	Leaves **/** Infusion	Fresh leaves infusion is taken orally for body cooling and against malaria. As it is bitter in taste, sugar is mostly eaten just after swallowing it to remove its bitterness, especially by kids.	No	No	47 (41)
*Thymus linearis* Benth. (127211) **Lamiaceae**	*marvai***/** Herb	Mountains **/** Frequent	Gastrointestinal	Oral **/** Fresh	Leaves **/** Infusion	Fresh leaves are cut into pieces, put in water, placed below stars for whole night, sieved and drunk early at the morning for stomach ailments.	[35, 42]	Yes	6 (6)
*Valeriana jatamansi* Jones (127214) **Caprifoliaceae**	*da dasthu botai***/** Herb	Mountains **/** Frequent	Gastrointestinal	Oral **/** Fresh	Leaves **/** Juice	Juice of fresh leaves are taken orally against diarrhea.	No	No	21 (20)
*Viburnum cotinifolium* D. Don (127210) **Adoxaceae**	*thoray***/** Shrub	Mountains **/** Frequent	Gastrointestinal	Oral **/** Fresh	Fruit **/** Without processing	Fruit is eaten fresh against constipation. **Food:** Common and well-known wild fruit of the area.	[43]	Yes	11 (8)
*Withania coagulans* (Stocks) Dunal (127195) **Solanaceae**	*khamazor***/** Shrub	Foothills **/** Frequent	Gastrointestinal (27), Multisystem (4), Hepatic (1)	Oral **/** Fresh & dried	Leaves (1), Fruit (31) **/** Infusion (5), Without processing (27)	Fresh or dried fruits infusion is taken orally to cool the body and against sunstroke. Fruits are taken with water (after removing exocarp) for abdominal pain, stomach problems and abdominal gasses. Young fruits are eaten directly to finish the effect of musculoskeletal ailments, also used against jaundice.	[36]	No	32 (32)
*Ziziphus jujuba* Mill. (127153) **Rhamnaceae**	*bera***/** Tree	Foothills **/** Occasional	Gastrointestinal	Oral **/** Fresh	Fruit **/** Without processing	Fruits are eaten fresh against constipation. It is not often eaten for this purpose but when eaten produces this effect. It is rarely available as its growing zone is below the foothills. **Food:** It is well known wild fruit of the area.	[44]	Yes	2 (2)
*Ziziphus oxyphylla* Edgew. (127199) **Rhamnaceae**	*heilaneiy***/** Tree	Foothills **/** Occasional	Respiratory (2), Gastrointestinal (1)	Oral **/** Fresh	Fruits **/** Without processing	Fruit is eaten fresh against cough and constipation. **Food:** It is well known wild fruit of the area.	Not reported against cough	Yes	3 (3)

^a^Wood-oil: It is obtained from burning the logs in a clavin-like structure and then collect it

Note: The numbers in column 4 and 6 are use reports

The no in inverted commas i.e. “No” in the second last column of the table shows not reported in the literature from the country for the culturally bound disease of the area i.e. ‘voice infection and masiyath’

### Preparation and application of medicinal plants

**Fig. 4:**
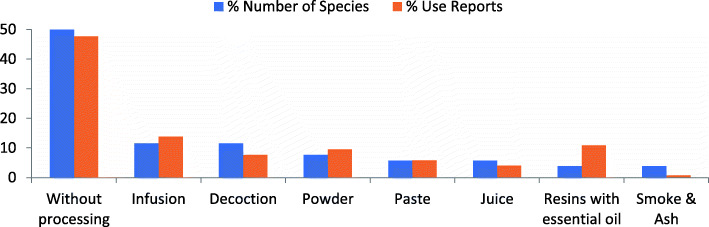
Various modes of preparation of medicinal plants

**Fig. 5:**
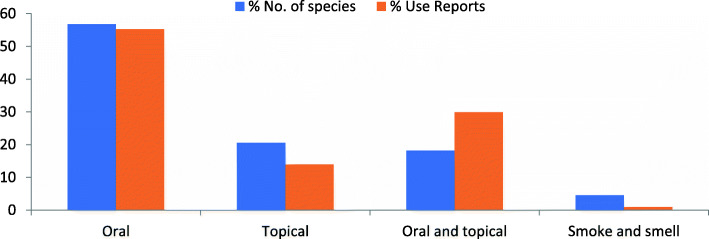
Route of administration of medicinal plant species

All the documented medicinal preparations are based on a single plant. Eight different ways of medicinal plant uses/preparations from different plant parts are employed. Most often, plant parts are used unprocessed (e.g., *Acacia modesta* and *Calotropis procera*), followed by infusion and decoction (Fig. 4). Medicine is taken orally (24 spp.) or used topically (9 spp.) and some species have both oral and topical applications (9 spp.). Only two species are used for their smell and smoke (Table 5, Fig. 5).

### Variation in knowledge among villages and informants

**Fig. 6:**
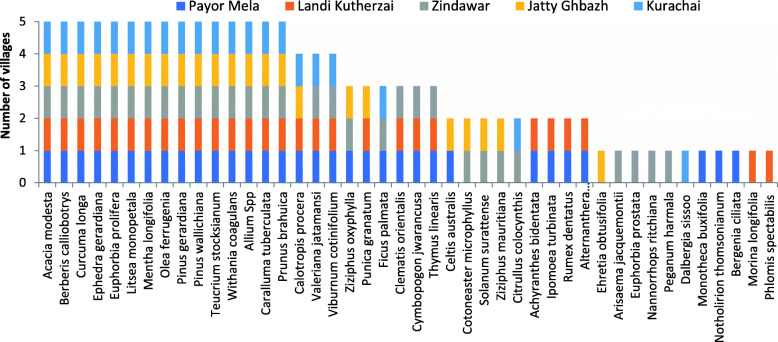
Medicinal plant species mentioned by informants of different villages

Medicinal plant knowledge is similar among villages (Fig. 6). Three-fourths of all medicinal plant species were mentioned in more than one of the villages, and 15 spp. were common to all villages’ informants. Also, sheep and goat skins are homogenously used in addition to animal and mineral based materia medica.

A comparison of age groups shows that informants between 20 and 29 years reported less medicinal plants than older people. Medicinal plant knowledge is transmitted vertically, i.e., from generation to generation and horizontally within the community especially among elders.

## Discussion

### Prevalent diseases and concepts

In the present study, gastrointestinal diseases have the highest number of species and use reports, followed by ritual uses and musculoskeletal ailments (number of use reports; Fig. 2). This mirrors the prevalence of diseases and treatments in the area. Gastrointestinal disorders are common usually due to contaminated water, which are preferably treated with medicinal plants. This pattern of treating gastrointestinal disorders with medicinal plants is found all over the world among rural communities and usually explained with the antimicrobial properties of many plants used as medicine [51–53]. Musculoskeletal problems are also common in the area due to the mountainous terrain and accident-prone livelihood activities such as carrying heavy commercial timber.

The category of ritual mainly covers treatments for diseases caused by spirits (Jinn), evil eye or ‘voice infection’. The concept of ‘voice infection’ could not be found in the available scientific literature, although it is deeply rooted in the local understanding of illness and is common in tribal areas of Pakistan and Afghanistan. In contrast, diseases caused by Jinn as well as the concept of evil eye is widely known in Islamic regions and broadly discussed in literature [54–56]. Medical doctors in the nearby area explained that the local concepts of “unusual diseases” seem to be related to epilepsy, psychological problems, and allergies.

Medicinal uses of plant species with a bitter taste like *Olea ferrugenia* fruits and *Caralluma tuberculata* aerial parts are based on the concept of ‘bitter taste is good for health’. This concept is found in many different cultures of the world [57–59].

Some diseases and medicinal plants are locally perceived as hot and cold, whereas the treatment is based on opposites. For example, malaria is considered a hot disease, and is treated with an infusion of *Teucrium stocksianum,* which is considered cold. Similarly, a *Withania coagulans* infusion is considered cold and is used against sunstroke, a locally perceived hot disease. Detailed knowledge about plant parts, preparations, and their degree of hotness and coldness is held and practiced by the herbalist/Hakim/Unani medicine specialists, which, however, is not found in our research area. A hot and cold dichotomy and the treatment with opposites is an integral part of the concept of humours and has been described for other regions of Pakistan [39, 60] and all over the globe [61–63].

### Local materia medica

The number of medicinal plant species reported from the Sulaiman area is less than reported by other studies, which typically report between 50 and 150 medicinal plant species for comparable sites [35, 37, 42]. This may have several reasons. Animal products, especially the use of goat and sheep skin, are of utmost importance for local treatments. Furthermore, a substantial part of the remedies is apotropaic and, in this case, often made from minerals or other products. In the Himalayan foothills of Southwest China, a similar situation was found with local healers—among the Shuhi people—who mainly work with ritual plants and their medicinal plant knowledge is relatively scarce compared to other regions [64]. In the Sulaiman area, health prevention through gathered wild food is also important and may be a reason for relatively little medicinal plant knowledge [31]. The numbers of species reported as ethnoveterinary [28] and edible plant species [31] were also less as compared to other areas. Apart from above-mentioned reasons, our research area has semi-arid climatic conditions which support comparatively less plant diversity; and—irrespective of most articles on medicinal plants from different parts of the country—we focused on a smaller area but with detailed documentation/evaluation.

All of the documented medicinal plant species are reported from other areas of Pakistan with similar or different uses [30, 36, 37, 40, 41, 65, 66]. Especially the ritual use of some of the species reported for problems like *‘voice infection’, masiyath*, and evil eye (rarely reported as bad eye), seem to be unique to the Sulaiman area and its local culture. Above half of species (52%) are new or unreported from the country for presently mentioned human ailments (Table 5). Same was the case for ethnoveterinary medicinal plants [28], whereas one-third species of the wild edibles were also newly reported from the study area [31], which shows the uniqueness of the Sulaiman area and its culture. About half of all use reports were from only 6 plant families, i.e., Lamiaceae (82 UR), Pinaceae (64 UR), Apocynaceae (62 UR), Euphorbiacaee (43 UR), Solanaceae (36 UR) and Rosaceae (23 UR). The relative importance of Pinaceae is due to two Pinus species (*P. gerardiana* and *P. wallichiana*) which are broadly used in the area not only for medicinal but also for ethnoveterinary medication and food purposes [28, 31]. The extent of similarity of these results with the prevalence of the families in the local vegetation is unknown, as a checklist of the flora of the Sulaiman Mountains is unavailable.

All reported medicinal plants are used individually without mixing different species or parts together during preparation, and the majority of medicinal plants are directly used without any prior processing/preparation (Fig. 4). There are different medicinal and ritual specialists, but no local herbalists in the area. This coupled with the predominant use of fresh plants, animals and minerals for medicinal purposes indicates that the traditional healing system consists of a combination of knowledge from different systems including biomedicine. A negative impact of syncretism between traditional and biomedicine is that local people tend to use pharmaceuticals like pain killers carelessly since they are unaware of possible side effects and proper dosage. These concepts are unknown to their traditional medicine. The local use of pharmaceuticals based on traditional concepts of plant medicine and related problems have also been described and discussed for two Amazonian societies [57].

**Fig. 7:**
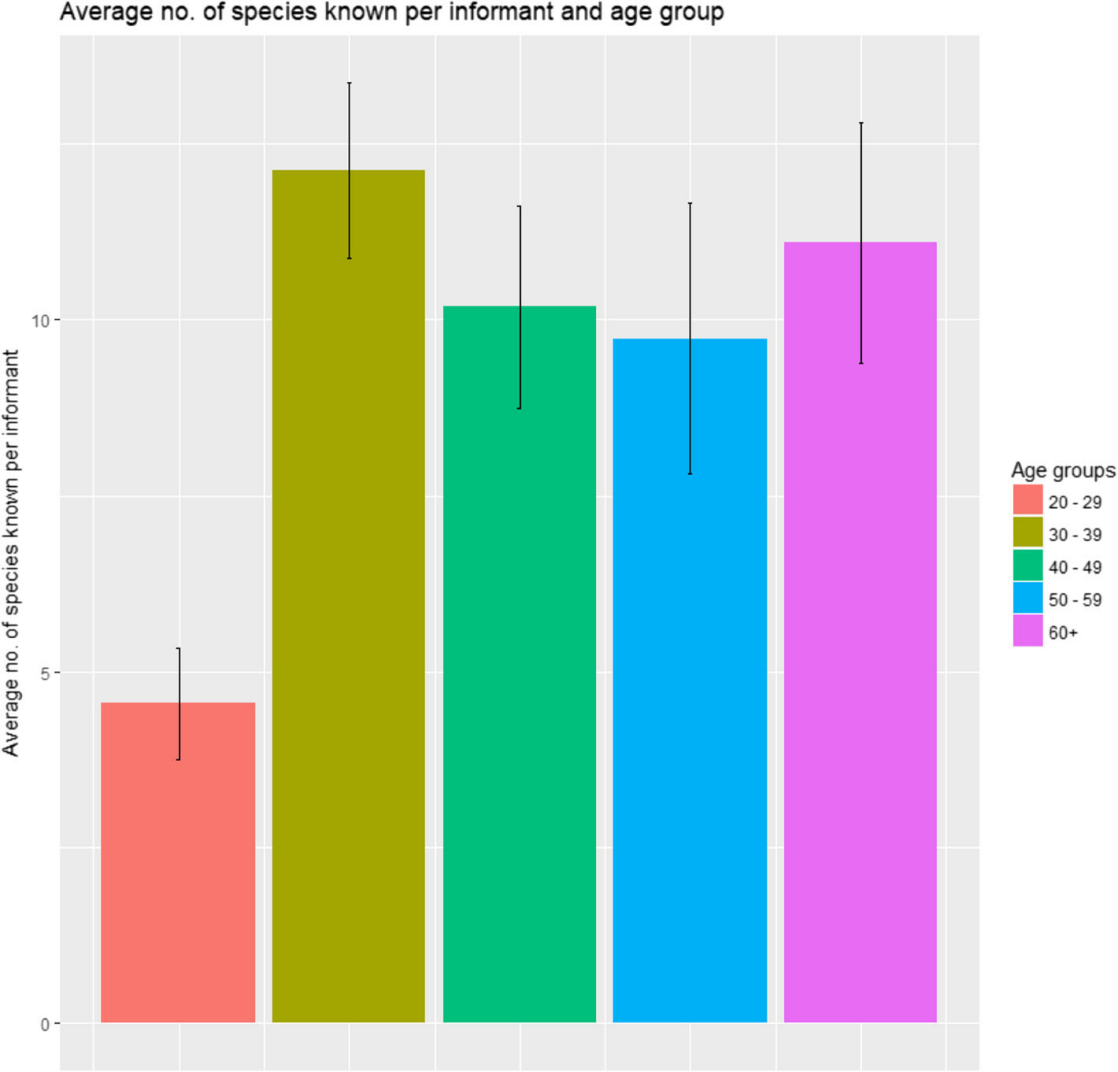
Age wise variations in medicinal plants knowledge among the inhabitants of the study area

Medicinal plant knowledge has some variations between villages (Fig. 6) possibly due to socio-economic differences (Table 1), weaker contacts (horizontal transmission of knowledge), and differences in their exposure to diverse flora [28, 31]. While age-wise knowledge difference (Fig. 7), is a universal phenomenon in traditional medicine, although, in our case it was ubiquitous in all age groups except the young (age 20–29). The commonality of medicinal plant knowledge was relatively more prevalent than the ethnoveterinary species [28] and wild edibles [31]. However, literature supports the commonalities of edible plants knowledge and use—as compared to ethnomedicinal ones—due to the sensitivity of health-related issues/knowledge [67]. Possible reasons include the strong culture of attending/taking care of patients by the neighboring villagers where they provide/share the best advice/knowledge, which is usually warmly welcomed by the patient’s family.

Local medicinal plant use is still dynamic. Some medicinal plants are used less recently while others are newly integrated into the materia medica. Plant medicine might be abandoned due to lack of efficiency, problems of availability, or cheap pharmaceutical alternatives. For example, there was a decrease in the use of *Phlomoides spectabilis* leaves against human skin allergy. Newly integrated species are *Valeriana jatamansi* against diarrhea, ca. 10 years ago, and *Thymus linearis* against stomach problems, ca. 15 years ago. Key informants claimed that the extensive use of few medicinal plants like *Teucrium stocksianum, Ephedra gerardiana* and *Withania coagulans* (Table 6)—compared to available pharmaceuticals—is due to their efficacy. Plants with high fidelity levels (e.g., *Valeriana jatamansi*, *Litsea monopetala*, *Berberis calliobotrys*, *Withania coagulans* and *Pinus* species-Table 6) are also used for similar ailments in livestock [28]. The globally increasing tendency of using traditional medicine may led to some harmful/poisonous or unpredictable side effects, e.g., several species mentioned during present study (*Euphorbia*, *Ephedra*, *Citrullus*, etc.) are reported in literature with adverse effects [38, 68, 69]. Therefore, official pharmacopeias must be consulted before using such plants or its parts.

**Table 6:** Most frequently used plants (*n* = 47) for different ailments with their fidelity level (FL)

Species	Disease	Specific use reports	Total use reports	FL (%)
*Euphorbia prolifera* Buch.-Ham. ex D. Don (127159) **Euphorbiaceae**	‘Voice infection’	42	42	100
*Valeriana jatamansi* Jones (127214) **Caprifoliaceae**	Diarrhea	21	21	100
*Litsea monopetala* (Roxb.) Pers. (127249) **Lauraceae**	Jaundice	18	18	100
*Viburnum cotinifolium* D. Don (127210) **Adoxaceae**	Constipation	11	11	100
*Teucrium stocksianum* Boiss. (127267) **Lamiaceae**	Malaria	46	47	97.8
*Mentha longifolia* (L.) L. (127209) **Lamiaceae**	Stomach problems and diarrhea	26	28	92.8
*Berberis calliobotrys* Bien. ex Koehne (127222) **Berberidaceae**	Musculoskeletal	24	27	88.9
*Ephedra gerardiana* Wall. ex Stapf. (127150) **Ephedraceae**	Stomachache	39	44	88.6
*Olea ferruginea* Wall. ex Aitch. (127151) **Oleaceae**	Constipation	28	32	87.5
*Withania coagulans* (Stocks) Dunal (127195) **Solanaceae**	Abdominal pain, stomachache, abdominal gasses	27	32	84.4
*Acacia modesta* Wall. (127146) **Fabaceae**	Musculoskeletal	11	14	78.6
*Prunus brahuica* (Boiss.) Aitch. & Hemsl. (127160) **Rosaceae**	Snake repellent	14	20	70
*Pinus wallichiana* A.B.Jacks. (127213) **Pinaceae**	Musculoskeletal	22	34	64
*Calotropis procera* (Aiton) Dryand. (127228) **Apocynaceae**	‘Voice infection’	16	25	64
*Curcuma longa* L. (127235) **Zingiberaceae**	Musculoskeletal	8	16	50
*Curcuma longa* L. (127235) **Zingiberaceae**	Jaundice	8	16	50
*Calotropis procera* (Aiton) Dryand. (127228) **Apocynaceae**	Wounds and abscesses	9	25	36
*Pinus gerardiana* Wall. ex D. Don (127203) **Pinaceae**	‘Voice infection’	10	30	33
*Caralluma tuberculata* N.E. Br. (127133) **Apocynaceae**	Diabetes	12	37	32
*Pinus gerardiana* Wall. ex D. Don (127203) **Pinaceae**	Wound healing	9	30	30

Extensive use of goat and sheep skin for medicinal treatment (Table 4) to our knowledge has not been reported in the ethnomedical literature of Pakistan yet. These uses are not restricted to the present research area but are typically found among Pashtun tribes in Pakistan and Afghanistan (personal discussion with residents of different areas including people of Afghanistan and Pashtun tribes of Pakistan). Similarly, the use of gold, silver and mineral stones against ‘voice infection’ is also practiced in the adjoining tribal areas (personal observation). These uses of materia medica are transmitted as oral histories. The uses of mineral stone (دہ ژاغ دانہ) against ‘voice infection’ are more important in off seasons when fresh plant material (especially *Euphorbia prolifera* with fidelity level 100%, Table 6) is scarce.

One of the reasons why the Sulaiman Markhor (*Capra falconeri*) appears as threatened species on the IUCN Red List 2016 is its high demand for medicinal purposes (e.g., bile of gall bladder for hepatitis, skin for multiple medicinal purposes and meat for general health support). Its horns are used for decoration, and both skin and horns fetch high prices in the market. Sustainable conservation strategy in the form of ecotourism and applying other conservation tools, by involving local communities—as they are familiar with vegetation, habitat and associated wildlife, needs to be devised in the area [43].

Interestingly, no medicinal plant trade is found in the research area. Some species like fruits of *Withania coagulance* and seeds of *Pegnum harmalla* are commonly marketed in Pakistan, even in the surrounding communities of the research area, but their prices are unenticing. Other plants like *Berberis calliobotrys, Ephedra gerardiana* and *Velariana jatamansi* have a high market demand and catch good prices [44], but locals were unaware about these commercial values. Sustainable harvesting of such plants could help to improve local livelihoods [70]. Above half of the present ethnomedicinal plant species were commonly available (Table 5), and leaves were the most used parts (Fig. 3), so were less critical for the subsistence needs of the locals. The priority must be given to the rarely available species with higher URs and FL (Table 5-6), because frequent uses decrease its availability. The ethnomedicinal knowledge in the area was also facing degradation- although not very high, which negatively affects the lives and culture of these societies.

## Conclusions

The present paper based on interactions with local informants investigates the traditional medicinal knowledge and materia medica of remote tribal communities in west Pakistan. A variety of medical substances from plants, animals and minerals are used to treat diseases, depending on the severity of the disease and availability of the substance. Treatment often happens in the family context. But different types of medical and ritual specialists are consulted if necessary, especially in the case of unusual diseases and illnesses caused by spirits. The local medicinal system is dynamic as it not only includes and integrates new medicinal plants but also pharmaceuticals. However, most important is the use of goat and sheep skin which forms a central pillar for healing. The use and practices mentioned during present study needs detailed pharmaceutical evaluation before its recommendation for general use. The widely used materia medica with rare availability needs conservation priority. Similarly, the local cultural norms are the means of matria medica practices which must be preserved. While the area faces some acculturation processes, traditional practices remain quite intact. From a developmental perspective, reinforcement of local institutional contexts would be important to strengthen local knowledge and related sustainable practices.

## Data Availability

The datasets generated and analyzed during the current study are not publicly available due to the easy identification of included participants. However, the corresponding author can be contacted to further explore the data, upon request.

## Abbreviations

FATA: Federally administered tribal areas
F.R. D.I: Khan- frontier region of Dera Ismail Khan
URs: Use reports
FL: Fidelity level
DEM: Digital elevation model
